# Finding tau rhythms in EEG: An independent component analysis approach

**DOI:** 10.1002/hbm.26572

**Published:** 2024-01-29

**Authors:** Matthew G. Wisniewski, Chelsea N. Joyner, Alexandria C. Zakrzewski, Scott Makeig

**Affiliations:** ^1^ Kansas State University Manhattan Kansas USA; ^2^ Swartz Center for Computational Neuroscience University of California San Diego La Jolla California USA

**Keywords:** auditory alpha, auditory perception, event‐related desynchronization, source localization, time‐frequency

## Abstract

Tau rhythms are largely defined by sound responsive alpha band (~8–13 Hz) oscillations generated largely within auditory areas of the superior temporal gyri. Studies of tau have mostly employed magnetoencephalography or intracranial recording because of tau's elusiveness in the electroencephalogram. Here, we demonstrate that independent component analysis (ICA) decomposition can be an effective way to identify tau sources and study tau source activities in EEG recordings. Subjects (*N* = 18) were passively exposed to complex acoustic stimuli while the EEG was recorded from 68 electrodes across the scalp. Subjects' data were split into 60 parallel processing pipelines entailing use of five levels of high‐pass filtering (passbands of 0.1, 0.5, 1, 2, and 4 Hz), three levels of low‐pass filtering (25, 50, and 100 Hz), and four different ICA algorithms (fastICA, infomax, adaptive mixture ICA [AMICA], and multi‐model AMICA [mAMICA]). Tau‐related independent component (IC) processes were identified from this data as being localized near the superior temporal gyri with a spectral peak in the 8–13 Hz alpha band. These “tau ICs” showed alpha suppression during sound presentations that was not seen for other commonly observed IC clusters with spectral peaks in the alpha range (e.g., those associated with somatomotor mu, and parietal or occipital alpha). The choice of analysis parameters impacted the likelihood of obtaining tau ICs from an ICA decomposition. Lower cutoff frequencies for high‐pass filtering resulted in significantly fewer subjects showing a tau IC than more aggressive high‐pass filtering. Decomposition using the fastICA algorithm performed the poorest in this regard, while mAMICA performed best. The best combination of filters and ICA model choice was able to identify at least one tau IC in the data of ~94% of the sample. Altogether, the data reveal close similarities between tau EEG IC dynamics and tau dynamics observed in MEG and intracranial data. Use of relatively aggressive high‐pass filters and mAMICA decomposition should allow researchers to identify and characterize tau rhythms in a majority of their subjects. We believe adopting the ICA decomposition approach to EEG analysis can increase the rate and range of discoveries related to auditory responsive tau rhythms.

## Graphical abstract

Auditory related alpha rhythms (i.e., tau rhythms) have historically been difficult to examine with the electroencephalogram (EEG). Here, we show that independent components analysis, combined with the right preprocessing routines, can be an effective way to examine tau rhythms in the EEG.

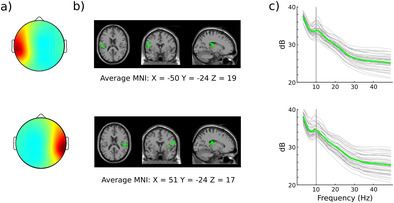

## 1 INTRODUCTION

### 1.1 Introduction

Research on alpha oscillations (~8–13 Hz) generated in visual, motor, and somatosensory areas of cortex has long been informing theory regarding the role oscillations play in information processing (e.g., Hanslmayr et al., 2011; Jensen & Mazaheri, 2010; Klimesch et al., 2007; Ross et al., 2022). Applications of this research include the development of brain computer interfaces that use alpha range oscillations as control signals (e.g., Stieger et al., 2021), and the monitoring/treatment of clinically relevant abnormalities in cortical activity (e.g., Deiber et al., 2020). However, auditory‐related alpha activity (i.e., tau rhythms) has been much less studied. We will use the tau terminology for the rest of this manuscript. The reason for the rarity of tau's study is not because it is less theoretically relevant than alpha localized to other brain regions. Theories proposing that alpha serves a functional role in perception have surfaced for auditory (Obleser & Kayser, 2019; Weisz et al., 2011) as well as other modalities (e.g., Buzsaki, 2006; Jensen & Mazaheri, 2010; Klimesch et al., 2007). The rarity of tau research is also unlikely to be linked to weak clinical relevance. Abnormally low alpha power within the superior temporal plane has been demonstrated in individuals suffering from tinnitus (Hartmann et al., 2014) and from schizophrenia (Koh et al., 2011). Rather, the reason for tau being understudied likely has more to do with the elusive nature of tau rhythms in scalp electroencephalographic (EEG) recordings. The current study represents initial development of an approach using independent component analysis (ICA) decomposition (Bell & Sejnowski, 1995; Makeig et al., 1996, 2002) to identify and measure tau rhythms in EEG data. Our motivation is the presumption that more readily available measures of tau EEG activity could greatly accelerate both basic and applied research on tau rhythms.

Unlike alpha oscillations generated in visual and somatomotor areas (mu rhythms), tau rhythms are not easily observable in “raw” scalp recordings in which stronger alpha rhythms originating in occipital and parietal cortex can easily mask them (Niedermeyer, 1991; Weisz et al., 2011). Early work using intracranial recordings from a single patient identified an auditorily responsive temporal alpha rhythm that was not apparent in visual inspection of the same patient's scalp EEG (Niedermeyer, 1991). Comparisons of magnetoencephalographic (MEG) with EEG data acquired using similar auditory paradigms have also led researchers to believe that EEG is not an appropriate imaging modality for studying tau (Bastarrika‐Iriarte & Caballero‐Gaudes, 2019). By consequence, tau rhythms have been most frequently studied using MEG or intracranial recordings where their presence is more easily demonstrated, perhaps reflecting in part the selectivity of MEG recording for sources originating in cortical tissue lying orthogonal to the brain/skull surface.

Tiihonen et al. (1991) presented subjects with sound sources of various duration and complexity and found stimulation‐reactive alpha band activity in the MEG. This alpha was suppressed during sound presentation and could be mapped to a source within the superior temporal plane. It could be distinguished from visual alpha and somatomotor mu rhythms in that it was not reactive to eye closure, body movements, or tactile stimulation (for related MEG work, see Bastarrika‐Iriarte & Caballero‐Gaudes, 2019; Frey et al., 2014; Hartmann et al., 2014; Lehtelä et al., 1997; Lorenz et al., 2009; van Dijk et al., 2010; Yokosawa et al., 2020). Recently, Billig et al. (2019) recorded alpha suppression following speech sound presentation from electrodes placed within Heschl's gyrus of neurosurgical patients (also, see Kumar et al., 2021). Baseline alpha power and sound‐induced suppression was evident in recordings from primary and secondary auditory areas.

Considered together, these MEG and intracranial results have convincingly demonstrated the existence of tau rhythms that can be distinguished from other cortical alpha rhythms. Knowledge development in this area has been stunted; however, by the fact that most researchers have at best limited access to MEG and intracranial recording. When these modalities are available, their use often necessitates a low sample size (e.g., when working with neurosurgical patients), are expensive to conduct (in the case of MEG), and are restrictive in terms of the types of experiments that can be conducted (e.g., in regard to movements, environment, etc.).

### 1.2 An ICA approach to tau rhythm analysis

Some researchers using an ICA decomposition approach to EEG analysis have identified independent components (ICs) of multichannel EEG data that appear to mimic the dynamics of tau as characterized with MEG and intracranial recordings. ICA decomposition learns linear spatial filters (linear combinations of the scalp channels) that jointly separate the recorded data into IC processes with fixed scalp data projection patterns (scalp maps) and time courses as temporally independent of one another as possible (Makeig et al., 1996). ICA as a blind source separation procedure is most popularly used as a way of identifying non‐brain source activities (artifacts, e.g., arising from eye blinks; Jung et al., 2001), and then removing them from scalp EEG recordings. However, there are substantial advantages to analyzing IC source activities themselves, especially those that by their activity characteristics and scalp projection patterns are compatible with sources generated in one, or sometimes two, presumably anatomically connected cortical areas (Delorme et al., 2012). The most relevant advantage regarding tau rhythms is that sources with temporally independent activities can be separated from one another despite having overlapping scalp projection patterns, producing their mixing in the scalp channel data (cf. Makeig et al., 1997, 2004; Onton et al., 2005).

Listeners in an auditory distance judgment task showed alpha suppression during presentation of sounds, the strength of which was associated with sound familiarity and distance judgment accuracy (Wisniewski et al., 2012, 2014). Much of this suppression was attributable to IC sources that could be localized to left and right superior temporal gyri. Furthermore, response‐time sorted analyses revealed that alpha suppression was aligned in time with stimulus onsets rather than manual button presses, supporting their distinction from sensorimotor processes (cf. the intracranial and MEG work cited above). Similarly, Jenson et al. (2015) identified left and right temporal ICs that showed alpha suppression during presentation of single phonemes (/*ba*/ and /*da*/). These ICs also showed enhancement during vocal production, while mu rhythm ICs showed suppression. This suggests that alpha activity in these sources was distinct from sensorimotor mu (also, see Bowers et al., 2019; Wisniewski & Zakrzewski, 2023). These studies introduce the exciting prospect that ICA decomposition may provide: (1) reliable characterization of tau activity in EEG data, increasing the pace of discovery through its wide availability, and (2) the capability to examine tau activity in more varied data collection scenarios, thereby increasing the range of discovery.^1^

There are at least two issues with the ICA decomposition approach to analysis of tau rhythms that need to be resolved to take full advantage of it. The first is that studies reporting tau ICs have only identified these ICs in a relatively small percentage of subjects. This “*sparsity problem*” hinders the ability to study tau ICs efficiently. For instance, Jenson et al. (2015) reported identifying left and right tau ICs for only 15 of 29 participants. Plöchl et al. (2016), after combining left and right lateralized ICs with dual‐dipole ICs (with bilaterally symmetric projection patterns), reported finding tau ICs in 8 of 14 participants. In earlier studies, we also found that roughly 50% of subjects in our samples exhibit this IC type (e.g., Wisniewski et al., 2012; Wisniewski et al., 2014; Wisniewski et al., 2021). Likely due to their comparative rarity in the data (and/or the dominance of visual perception studies in the literature), only a fraction of studies using ICA decomposition have reported finding tau ICs. ICs accounting for other EEG rhythmicities are much more commonly found and reported (e.g., frontal midline theta, mu rhythm, occipital and parietal alpha rhythms). One possibility is that the sparsity problem is exacerbated by use of suboptimal analysis parameters. The type of ICA decomposition approach and the filter settings used in preprocessing can strongly impact resulting ICA decompositions (Delorme et al., 2012; Hsu et al., 2018). To date, there have been no studies examining how these parameters impact the likelihood of obtaining tau ICs from an ICA decomposition.

A second issue is that the separation of tau rhythm source alpha dynamics from other brain activities has only been tentatively established. We will refer to this as the “*distinctiveness problem*.” In more complex paradigms, the dynamics of multiple IC processes can show parallel frequency modulations. For instance, we recently reported that left tau, parietal alpha, and occipital alpha all showed power suppression during the presentation of target speech in a speech‐on‐speech masking task (Wisniewski et al., 2021). In MEG and intracranial research, studies have in several cases employed passive audio presentation paradigms so as to minimize the influence of the many brain rhythms associated with more complex tasks (e.g., somatomotor mu or occipitoparietal alpha rhythms). To our knowledge, a study has not been conducted to assess how best to isolate tau ICs in EEG data recorded in a passive presentation paradigm.

### 1.3 Current study

Here, we address how choices in EEG data processing impact the likelihood of identifying cortical source‐compatible ICs that account for tau activity in the scalp EEG (addressing the sparsity problem), and how such source‐resolved tau IC dynamics relate to the alpha band dynamics of other brain‐source ICs (addressing the distinctiveness problem). We exposed listeners to complex non‐speech sounds while the EEG was recorded using a 68‐channel scalp electrode montage. The artifact‐rejected EEG data were copied into several EEGLAB datasets that were then processed using one of five high‐pass filters (0.1, 0.5, 1, 2, and 4 Hz), three low‐pass filter settings (25, 50, and 100 Hz), and four ICA decomposition algorithms—fastICA, extended infomax ICA, adaptive mixture ICA (AMICA), and multi‐model adaptive mixture ICA (mAMICA). This resulted in 5 × 3 × 4 = 60 separate ICA decompositions of the recorded EEG data for each subject.

The data unmixing matrix produced by each ICA decomposition was applied to that subject's original artifact‐rejected data. ICs were then automatically classified as most likely arising from brain, eye, muscle, line noise, or “other” effective sources using ICLabel (Pion‐Tonachini et al., 2019), and those suggested by ICLabel to be brain‐based were then localized using single equivalent current dipole models applied to their scalp maps. After this, we computed brain‐based IC mean log spectra and sound presentation event‐related spectral perturbations (ERSPs). Brain‐based ICs were blindly clustered within each set of analysis parameters. We anticipated that clusters of left and right tau ICs would be revealed and that some analysis pipeline/method choices would yield more ICs within these clusters than others. Specifically, we hypothesized that the best combination of parameters would yield a larger percentage of contributing subjects than previously reported (>50%). If this hypothesis was confirmed, we and others might use those parameters to optimize results of ICA decomposition of data from auditory tasks. We further expected that tau IC clusters would show a characteristic alpha suppression during passive sound presentations that was distinguishable from presentation‐related spectral dynamics of IC sources accounting for somatomotor mu, parietal, and occipital alpha activities. This would suggest that the characteristics of tau IC sources parallel those of tau as examined using MEG or intracranial recordings.

## 2 METHODS

### 2.1 Participants

Procedures were approved by the Kansas State University ethics committee. Twenty‐five individuals from undergraduate courses at Kansas State University or from the local community participated. In exchange for their participation they received course credit or payment of $15/h. All individuals reported normal hearing. This study was conducted during the COVID‐19 pandemic in which safety protocols entailed constant wearing of face masks and the absence of active ventilation in our sound attenuating booth. Seven individuals' data were contaminated by high‐amplitude, low frequency skin potentials, or excessive movement due to their sensitivity to these unique recording circumstances. Data of these individuals were dropped from analysis.

### 2.2 Equipment

All stimuli were presented over Etymotic ER‐2 earphones (Etymotic, Elk Grove Village, IL) connected to an RME Fireface UC audio interface (RME‐audio, Germany). Volume levels were set such that stimuli were presented at ~79 dB SPL. Listeners sat in a WhisperRoom sound attenuating booth throughout the study (WhisperRoom, Knoxville, TN). For EEG recording, a 68‐channel Biosemi Active II setup was used (see below for more detail). Stimulus timing was monitored by recording the output of the audio interface together with the EEG using a Biosemi analog input box.

### 2.3 Stimuli

**FIGURE 1:**
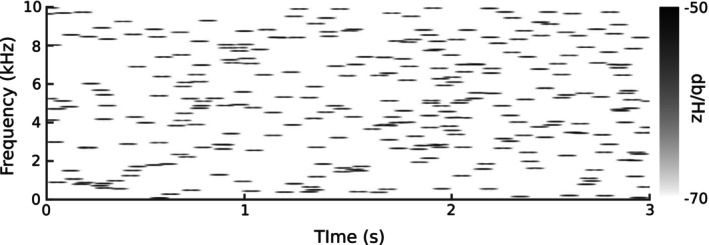
Spectrogram of an example asynchronous tone train.

Sound stimuli were asynchronous tone trains 3 s in duration, with each tone in the train being of 50 ms duration including 10‐ms cosine ramped on‐ and off‐sets. The density of tones within these trains was 300 per train (~100 tones per s). Figure 1 gives a spectrogram for an example train. Asynchronous tone trains were used because single unit recordings in nonhumans have shown that such stimuli yield stronger responses in auditory cortex compared to simple pure‐tone stimuli (DeCharms et al., 1998). Further, individuals hearing asynchronous tone trains are not likely to have pre‐associated meanings or biases related to these sounds as they would for speech or other ecologically meaningful stimuli.

### 2.4 Procedures

Participants were asked to bring reading material of their choosing to the experimental session. They were instructed to read this material and to ignore the sounds being presented throughout the experiment. They were also instructed to remain still. No other task was given.

There were three types of blocks with different types of sound exposure: no‐sound, diotic, or dichotic. All blocks were 60 s in duration. In no‐sound blocks, no sounds were presented. This served to give subjects a break from constant stimulation conditions. In diotic presentation blocks, the 3‐s tone trains were presented separated by 2‐s inter‐stimulus intervals (ISIs). In the dichotic blocks, the same tone trains were presented with the same ISIs, but tone train presentations alternated between the left or right ears. There were five blocks of each type. This produced a total of ~60 tone train presentations in the diotic and dichotic conditions. Initial analyses indicated no difference in the likelihood of obtaining a tau IC across these different spatial conditions. We therefore collapsed across all diotic and dichotic blocks in the analyses we report here.

### 2.5 EEG acquisition

A cap with 64 electrode wells was fitted onto participants' heads (see https://www.biosemi.com/download.htm for specific electrode coordinates). Additional electrodes were placed lateral to each eye, and on each mastoid. Individual electrode locations were then recorded using a Polhemus Patriot 3D digitizing system (Polhemus, Colchester, VT). Electrodes were then placed within electrode wells of the cap which were pre‐filled with conductive gel.

Recordings were referenced online to the common‐mode‐sense/driven‐right‐leg (CMS/DRL) of the Biosemi system. Electrode offsets were brought within 25 μV of CMS/DRL or else were rejected from analysis. The EEG was subsequently recorded at a 2048 Hz per channel sampling rate with 24‐bit A/D resolution.

### 2.6 Initial data processing and parallel analysis pipelines

**FIGURE 2:**
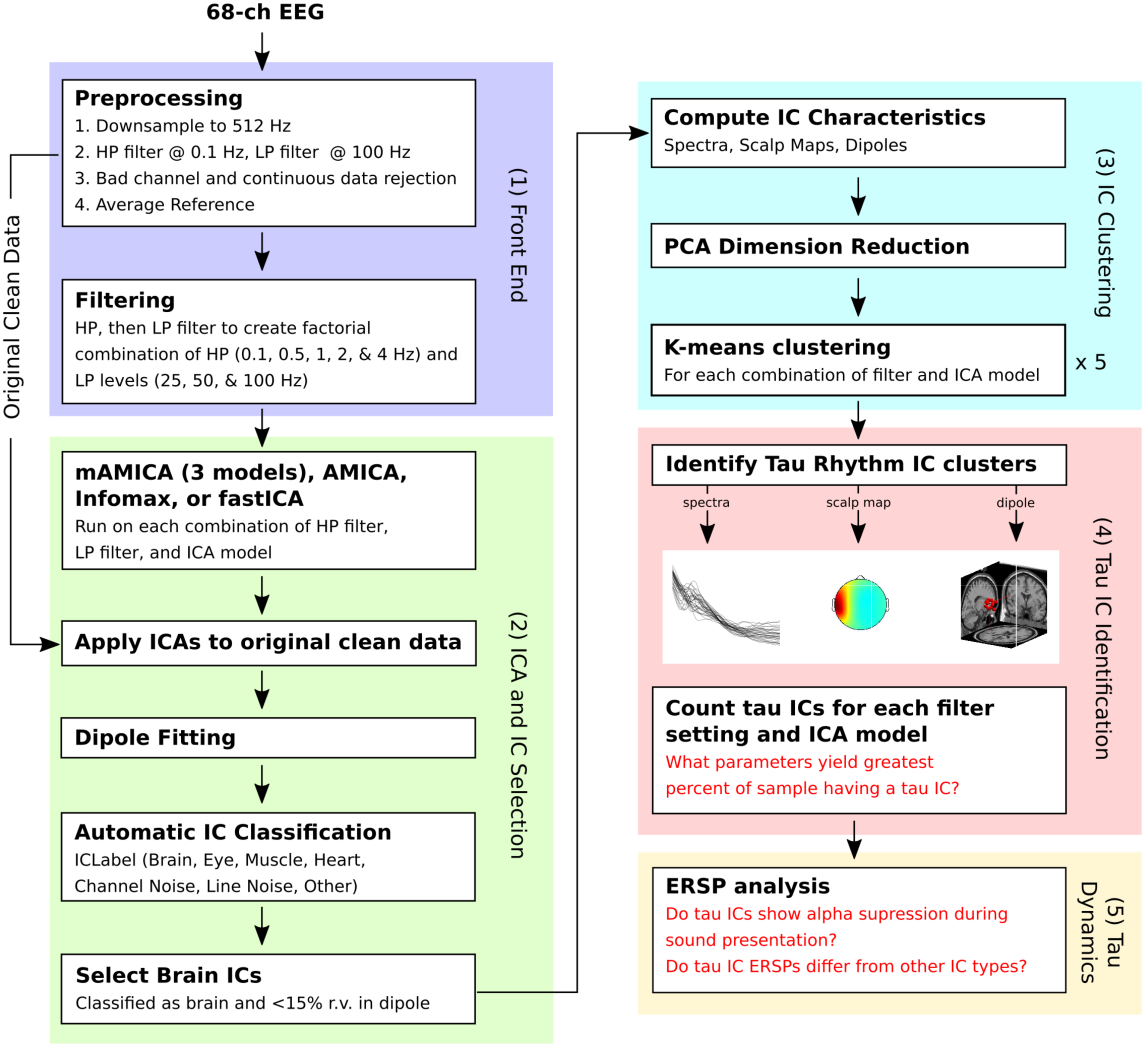
Depiction of the analysis pipeline.

The analysis pipeline described below is depicted in Figure 2. All offline analyses were performed using EEGLAB (Delorme & Makeig, 2004; https://sccn.ucsd.edu/eeglab/index.php), and custom MATLAB scripts. The data were re‐referenced offline to average reference (removing the influence of the DRL circuit from the data), resampled at 512 Hz (after applying an antialiasing filter), digitally high‐pass filtered (16,897‐point zero‐phase FIR, 0.1‐Hz passband edge), then low‐pass filtered (69‐point zero‐phase FIR, 100 Hz passband edge). Channels and portions of the continuous data determined by visual inspection to be contaminated by excessive noise were then removed. After this initial preprocessing, each subject had a base dataset that was preserved for later unmixing using the ICA weights learned by the 60 analysis pipeline variants.

Separate datasets were then created after additional filtering corresponding to a factorial combination of high‐pass filter passband edges of 0.1, 0.5, 1, 2, and 4 Hz, and low‐pass filter passband edges of 25, 50, and 100 Hz. For combinations where passband edges were consistent with the original dataset, the additional high‐pass and/or low‐pass filtering was skipped. All filters were zero‐phase FIR. Details on half‐amplitude cutoff, transition band, and filter length (i.e., sample points) are given in Table 1.

**TABLE 1:** EEG filter details.

	Half‐amplitude cutoff	Transition band width	Points
*HP passband edge*			
0.1 Hz	0.05 Hz	0.1 Hz	16,897
0.5 Hz	0.25 Hz	0.5 Hz	3381
1 Hz	0.5 Hz	1 Hz	1691
2 Hz	1 Hz	2 Hz	847
4 Hz	3 Hz	2 Hz	847
*LP passband edge*			
25 Hz	28.125 Hz	6.25 Hz	273
50 Hz	56.25 Hz	12.5 Hz	137
100 Hz	112.5 Hz	25 Hz	69

*Note*: Sample rate is 512 Hz.

Abbreviations: HP, high‐pass; LP, low‐pass.

In general, ICA models find a set of weights (**W**) that linearly unmix the channel EEG data (**x**) into a sum of maximally temporally independent and spatially fixed components (**u**) such that **u** = **Wx**. The advantages of ICA are that the resulting component activities (rows of **u**) can be analyzed in the same manner as channel data (e.g., event‐related changes in alpha power can be determined), or removed from channel data if they are determined to reflect data artifacts (e.g., eyeblinks). The columns of the inverse matrix, **W** ^−1^, specify the spatial projection of each IC to the scalp channels. These projection maps can then be used to fit a single equivalent current dipole model for that component. For many ICs, a single equivalent dipole model can account for a large majority of the variance in the IC scalp projection pattern (scalp map) (Delorme et al., 2012).

There are several different types of ICA algorithms that vary in how they derive ICs. Though they converge on similar solutions, some ICA models work better than others (e.g., by finding more brain‐related ICs or identifying ICs that share less mutual information; Delorme et al., 2012). Here we used fastICA (Hyvärinen & Oja, 2000), infomax ICA (Bell & Sejnowski, 1995), AMICA (Palmer et al., 2008), and mAMICA (Hsu et al., 2018; Palmer et al., 2008). These were chosen because they are among the most frequently used ICA models applied to EEG data and/or have been shown to produce a large number of ICs resembling the projection of locally coherent cortical field potentials compared to others (e.g., as is the case for AMICA; Delorme et al., 2012). After training each ICA model, the trained ICA weights were then applied to the individual's baseline dataset.

The IC scalp projection maps were fit with equivalent current dipoles using the DIPFIT plug‐in for EEGLAB. Individualized recordings of electrode location (excluding the eye channels) were warped to a four‐layer MNI head model. Then a best‐fitting single equivalent current dipole was determined through a grid‐based search of the entire brain volume, followed by an iterative adjustment of this dipole to maximize the variance accounted for in the scalp projection of the IC. ICs were classified automatically as likely originating in brain, eye, muscle activity, line noise, or “other” using the ICLabel plug‐in for EEGLAB (Pion‐Tonachini et al., 2019). Any ICs estimated as having over 50% likelihood of originating in the brain itself, and having a single equivalent current dipole model explaining at least 85% of the variance in its scalp projection map were retained for later analyses.

### 2.7 Identification of tau IC clusters

The identified “brain ICs” were then blindly clustered based on their PCA‐reduced spectra (15 dimensions), PCA‐reduced scalp maps (15 dimensions), equivalent dipole location, and dipole moment. K‐Means was used to group ICs into 16 clusters, with ICs further than 3 SDs from any cluster centroid placed in a separate “outlier” cluster. All clustering was done independently for each combination of ICA algorithm and filter settings so as to best mimic a standard study where only one set of analysis/processing parameters is utilized. For some ICA/filtering combinations, IClabel applied in the previous step identified so few “brain” ICs, that it was not feasible to perform this clustering. When this was the case, that combination was considered to have no tau IC cluster. Different clustering iterations can produce different results, even when using identical clustering parameters. To address this issue, we repeated clustering five times for each combination of ICA/filtering parameters and saved the results of each for later analyses.

The process of identifying tau rhythm IC clusters was accomplished through selection of IC cluster scalp maps representing left and right tau based on their similarity to tau reported in the previous work of Wisniewski et al. (2012, 2021), Jenson et al. (2015), and Plöchl et al. (2016). A processing script was put together that cycled through presentations of images showing each set of IC cluster scalp maps, for each ICA/filtering combination, for each iteration of clustering. Scalp maps were numbered in the images, and the numbers were selected that were most similar to previous tau IC scalp maps. It was also possible to abstain from selection of a cluster if no map appeared to match previous tau IC reports.

In the initial presentation of these identified tau IC clusters, we present mean IC scalp maps, cluster equivalent current dipole centroids, and cluster spectra. The ICs contributing to tau clusters were then analyzed to determine the percent of the sample that contributed to the cluster. In plots, we report the mean values for percent of sample across the five clustering iterations. It was expected that these plots would reveal what combination of ICA/filtering parameters would optimize the identification of tau ICs. We statistically compared filter settings and ICA models using a permutation‐based procedure. High‐pass filter setting, low‐pass filter setting, and ICA model were assessed separately. Here, we will give an example for high‐pass filter paired comparisons, but the same procedure was used for low‐pass filter and ICA model paired comparisons. For each paired comparison, 1000 iterations permuting the high‐pass filter “label” for each subject was randomly determined. For example, for the 0.5‐Hz versus 1‐Hz comparison a subject's mean contribution to a tau cluster (averaged across all low‐pass filter settings and ICA models) for their 0.5‐ and 1‐Hz filtered data could be labeled either as 0.5 and 1 Hz or 1 and 0.5 Hz, respectively. The percent of sample contributions were recomputed for each iteration, creating a null hypothesis distribution of the difference between conditions expected by chance. A significant difference in the actual data was considered to be a difference in which no more than 0.8% of the null hypothesis distribution exceeded the observed value (i.e., *p* < .05 after Bonferroni Correction for each set of paired comparisons).

### 2.8 Tau IC dynamics

A single combination of analysis parameters was selected for further characterization of tau IC dynamics. This selection was based on the percent of sample data for left and right tau ICs. Data corresponding to the parameters yielding the largest percent of sample was chosen. In our data, this corresponded to a high‐pass filter passband of 4 Hz, low‐lass filter passband of 25 Hz, and the mAMICA algorithm. Epochs were made containing the −1.5 s to 4.5 surrounding the onset of the asynchronous tone trains. Event‐related spectra were computed for ICs using complex Morlet wavelets having 3 cycles at the lowest frequency (3 Hz), to 10 cycles at the highest frequency (50 Hz). This used the *newtimef*() function in EEGLAB. Resulting time‐frequency representations had time steps of ~14 ms and frequency steps of 0.5 Hz. A log mean baseline power spectrum between 500 and 100 ms preceding stimulus onsets was then removed to create ERSP images (Grandchamp & Delorme, 2011; Makeig, 1993). ERSP images show baseline relative power in decibels. ERSPs were examined for tau, occipital alpha, mu, and parietal alpha IC clusters that were identified from this single set of parameters.

To avoid interpretation bias arising from differences in subject contributions to IC clusters, we included only the single IC within a cluster for a subject that had the lowest variance rank (i.e., the IC that contributed the largest signal to the scalp data in relation to other ICs). This procedure mitigates the potential problem of one subject distorting interpretation by having multiple ICs contributing to a cluster‐mean ERSP image. Single‐subject significant changes in power from baseline were evaluated by shuffling time points to create a null hypothesis distribution for each time‐frequency point of the dB power values expected by chance. Any point at which less than 1% of the values in the null hypothesis distribution exceeded that of the real data was considered significant at this level. These single‐subject ERSPs were significance masked and then averaged across subjects. These average images where then further masked using a binomial test with an alpha level of 0.01 (cf. Onton et al., 2005).

## 3 RESULTS

### 3.1 Tau IC clusters

**FIGURE 3:**
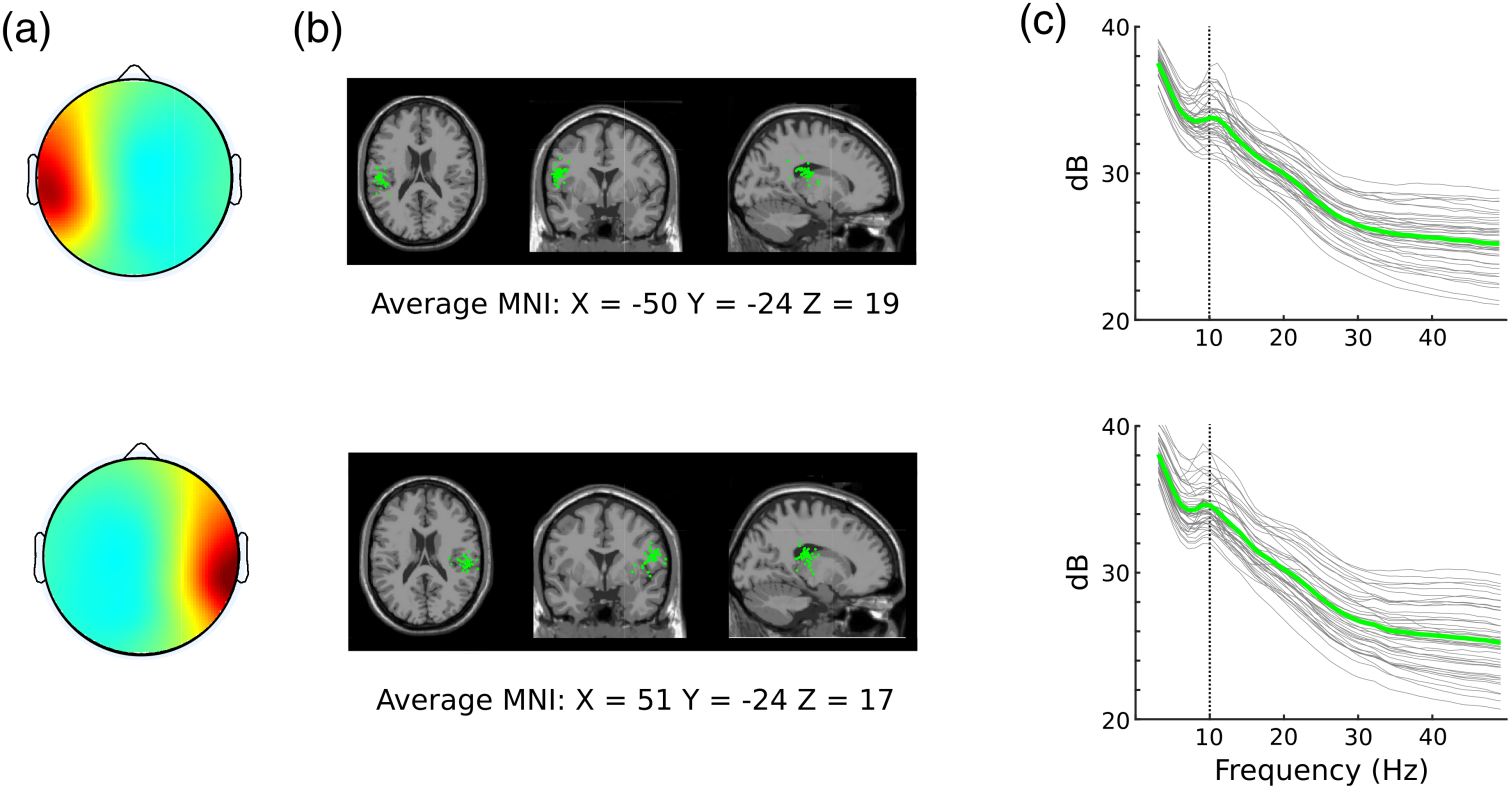
(a) Mean scalp maps for left (top row) and right (bottom row) clusters identified to contain tau independent components (ICs). Maps represent the average of all analysis settings and iterations of K‐means clustering. Note that the average maps were made by averaging the mean map of each cluster. Those original cluster maps had varying numbers of ICs and subjects. For these, and other IC scalp maps, coloring represents the inverse of weights for the IC returned by ICA. Because direction of weights is arbitrary, any ICs with strong negative polarities were flipped before averaging. (b) Locations of the centroid of IC modeled dipoles for each cluster of tau ICs. Each green sphere is a separate centroid plotted within the standard Montreal Neurological Institute (MNI) brain image with neurological convention (individual's right on right side of image). The grand average coordinates of these centroids are given. (c) Mean spectra for clusters of left and right tau ICs. The individual thin gray lines show spectra from a single set of analysis settings and iteration of clustering. The thick green line shows the average across all clusters. A line for 10 Hz is plotted for reference.

Figure 3 shows mean scalp maps, modeled dipoles, and spectral characteristics for IC clusters identified as tau‐related. In general, scalp maps are consistent with ICs previously claimed to be tau‐related (e.g., Jenson et al., 2014; Plöchl et al., 2016; Wisniewski et al., 2021), modeled equivalent current dipoles are near the superior temporal gyri (Billig et al., 2019), and spectra show peaks in the alpha band.

In Figure 3b, individual equivalent current dipoles are represented by small green spheres. Across clusters, centroids fell mostly within the inferior portion of the supramarginal gyrus, with some centroids in the superior temporal gyrus. Average MNI coordinates for left and right tau IC clusters were X = −50, Y = −24, and Z = 19 and X = 51, Y = −24, and Z = 17, respectively. It is notable that these coordinates lie within the supramarginal gyri for both clusters. Though this is the case, it has been demonstrated that equivalent current dipole modeling for temporal sources tends to yield results skewed toward superior locations (i.e., higher Z coordinates; Akalin Acar & Makeig, 2013). In that work, error was found to be 9.4 mm on average in the same four‐layer MNI model we used here. The standard template position of auditory cortex is within this margin of error for both left and right tau centroids.

The spectra for both left and right tau clusters show clear peaks in the (8–13 Hz) alpha range, consistent with intracranial and MEG recordings of the tau rhythm. Some of the clusters show beta band spectral peaks that accompany the lower alpha band peaks. Alpha‐harmonic peaks are well known to be associated with markedly non‐sinusoidal somatomotor mu‐rhythm waveforms. After identifying the filter and ICA model combination that discovers tau ICs in the data of the largest portion of the sample, we consider the separation of mu and tau components (see Section 3.2).

### 3.2 Filter settings and ICA model impact the likelihood of obtaining tau ICs

**FIGURE 4:**
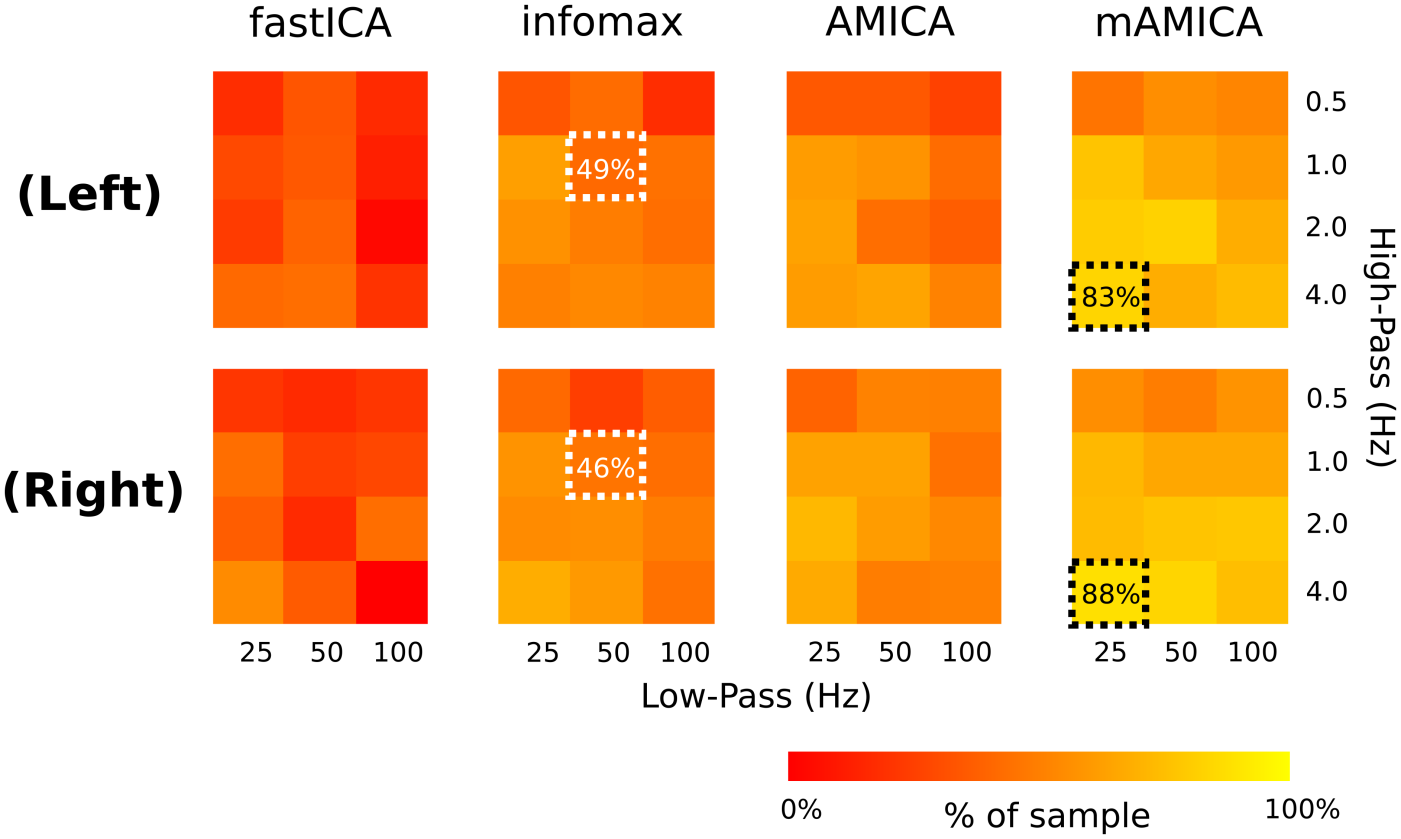
Percentages of subjects contributing to tau IC clusters in each combination of ICA algorithm and high‐ and low‐pass filter settings. The data reflects mean proportion of subjects across five iterations of IC clustering. White boxes outline a typical set of parameters (HP = 1 Hz, LP = 50 Hz, ICA model = infomax; cf. Wisniewski et al., 2021). The black boxes outline a better performing combination of parameters (HP = 2 Hz, LP = 50 Hz, ICA model = mAMICA). The percentages shown are rounded to the nearest integer.

Figure 4 depicts the percentage of subjects populating each of the tau IC clusters for each combination of high‐pass filter, low‐pass filter, and ICA model. High‐pass filter settings at 0.1 Hz yielded very few “brain” ICs under any low‐pass or ICA model combination. Thus, an HP of 0.1 Hz is omitted from the figure. The figure reflects the mean percentage obtained across the five iterations of clustering. In Figure 4, we also highlight an example of filter and ICA model combinations that is typical based on common use in other studies (white box) and a better performing atypical combination of settings (black boxes). Note that the percentage of the sample contributing to tau IC clusters under the typical combination are here similar to that reported previously; ~49% for left tau, ~46% for right tau. Other combinations of filter and ICA model choice obtain higher percentages obtained (>80%).

It is clear that relatively low HP filter passbands hinder the ability of ICA models to identify tau ICs. That is, the 0.1‐Hz high‐pass cutoff and the 0.5‐Hz high‐pass cutoff row gave low percentages of decompositions including a tau IC. This was expected as previous research has shown that ICA is most effective at identifying plausible brain‐related ICs when low frequency, high‐amplitude fluctuations in the EEG signal are filtered out (e.g., Klug & Gramann, 2021). Low‐pass filtering may not matter as much as high‐pass filtering, though there is some hint in these results that using a relatively high cutoff frequency (100 Hz) might give a smaller percentage of the sample having tau ICs than using lower cutoffs (25 or 50 Hz). The ICA algorithm used in the decomposition also appears to matter. FastICA identified fewer tau ICs than any of the other models, whereas the best performing ICA model was here mAMICA.

To assess impacts of filter and ICA parameters more formally, a permutation‐based procedure was used to test paired comparisons between each high‐pass filter, low‐pass filter, and ICA model setting (see Section 2.7 for detail). The results are summarized in Table 2, which reports the differences in percent of decompositions contributing to left and right tau clusters for each comparison. This analysis backs up the visual interpretation of Figure 4. First, decomposed data with relatively low high‐pass filter cutoffs tend to include fewer tau ICs than data with higher cutoffs, while comparison of low‐pass filter cutoffs failed to find any significant difference between low‐pass filter settings. mAMICA decomposition produced significantly more tau ICs than the other decompositions, while fastICA decomposition returned significantly fewer. No significant difference in percentage of tau ICs was observed between infomax ICA and single‐model AMICA decomposition. The latter two results are compatible with findings in (Delorme et al., 2012), while the first appears consistent with the general sensitivity of mAMICA to non‐stationarity in IC maps and activities (Hsu et al., 2018; Palmer et al., 2008).

**TABLE 2:** Impact of filter parameters and ICA model on percent of the sample contributing at least one IC to the left and right tau clusters.

	Left tau		Right tau	
	Difference	Sig.	Difference	Sig.
*High‐pass paired comparisons*				
0.5–1 Hz	−14.8	*	−12.8	*
0.5–2 Hz	−14.9		−17.2	***
0.5–4 Hz	−20.9	*	−17.5	
1–2 Hz	−0.1		−4.4	
1–4 Hz	−6.1		−4.7	
2–4 Hz	−6.2		−0.3	
*Low‐pass paired comparisons*				
25–50 Hz	1.21		1.21	
25–100 Hz	2.68		1.11	
50–100 Hz	1.48		−0.09	
*ICA model paired comparisons*				
fastICA—infomax	−19.3	*	−19.9	*
fastICA—AMICA	−21.9	*	−26.9	**
fastICA—mmAMICA	−41.4	***	−41.5	***
Infomax—AMICA	−2.6		−6.9	
Infomax—mmAMICA	−22.1	*	−21.57	***
AMICA—mmAMICA	−19.5	***	−14.6	**

*Note*: High‐pass cutoffs of 0.1 yielded to few “brain” ICs for cluster. Thus, they are left out of this analysis. All values reflect differences in % of sample yielding a left or right Tau IC. In computing percent of sample, the mean as taken across all iterations of cluster and across all combinations of the untested analysis parameters (e.g., when testing for high‐pass cutoff differences, the mean percent of sample was taken across all low‐pass cutoffs and ICA models). Asterisks mark significance as determined by Bonferroni corrected *p*‐values obtained through permutation testing.

* *p* < .05.

** *p* < .01.

*** *p* < .001.

**FIGURE 5:**
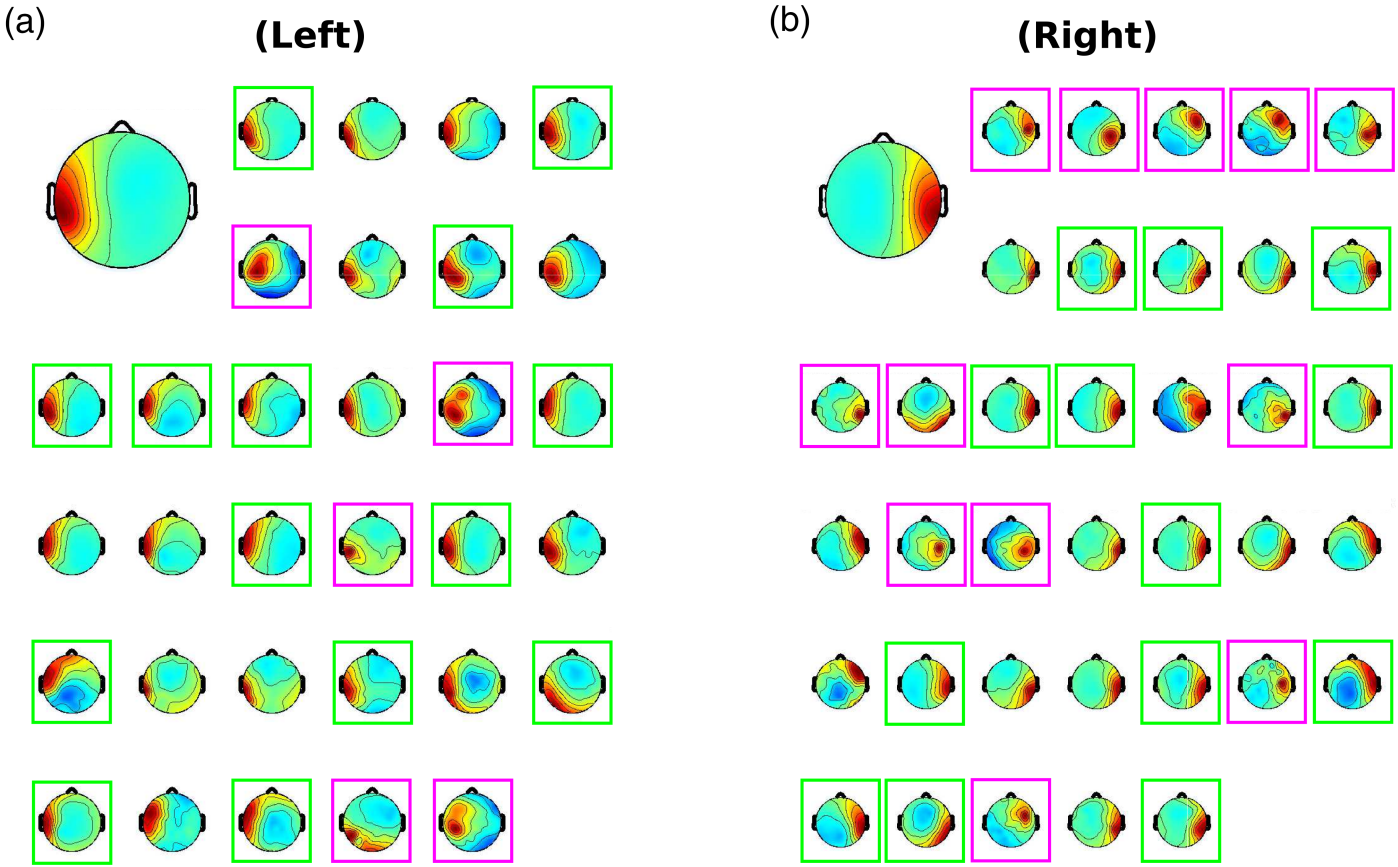
Individual independent component (IC) scalp maps for (a) left and (b) right tau IC clusters as discovered in one K‐means clustering iteration with a high‐pass filter setting of 4 Hz, a low‐pass filter setting of 25 Hz, and using the multi‐model adaptive mixture independent component analysis (mAMICA) model. Ordering is arbitrary, only reflecting the order of subject participation across the study. The large scalp maps show the average map across all ICs in each cluster. The green colored boxes highlight ICs forwarded to further analysis. The pink boxes highlight ICs thought unlikely to be tau‐related. Note that though average scalp maps appear to be mostly consistent with a radial source, that the individual scalp maps for tau components can vary.

To address the possibility that the identified tau rhythm clusters may have contained nonhomogeneous components, some of which might be better considered to represent non‐tau, for example, sensorimotor mu rhythms, we examined individual IC scalp maps for clusters corresponding to mAMICA trained on the 4‐Hz high‐pass, 25‐Hz low‐pass filtered data. Figure 5 shows individual scalp maps for each cluster IC. ICs appearing to depart from expected scalp maps are highlighted with pink boxes. Notably, several scalp maps consistent with mu rhythm ICs were clustered among the tau ICs. Inspection of the spectra of these ICs also revealed a typical mu rhythm profile with a beta harmonic peak accompanying an alpha peak (e.g., Delorme et al., 2012; Ross et al., 2022). There were also ICs seemingly better representative of electrode artifacts (with strong projection at a single electrode) or occipital alpha ICs. Nevertheless, even after eliminating these ICs from each cluster, a large percentage of the sample was retained for each: ~78% for the left tau cluster, ~72% for the right, with ~94% of the subjects contributing to either the left or right tau IC cluster.

We moved next to examine the spectral dynamics of these ICs to determine whether they mimic the alpha suppression of tau rhythms as observed with intracranial and MEG modalities. All these subsequent analyses were performed on data high‐pass filtered at 4 Hz, low‐pass filtered at 25 Hz, and trained with mAMICA. We only included those ICs that were the lowest ranked in terms of contribution to the channel data for each subject. That is, a subject contributed at most one IC to the analyzed left and right tau clusters. Scalp maps for these ICs are highlighted by green boxes in Figure 5.

### 3.3 Tau ICs show alpha suppression during sound presentation while other ICs do not

**FIGURE 6:**
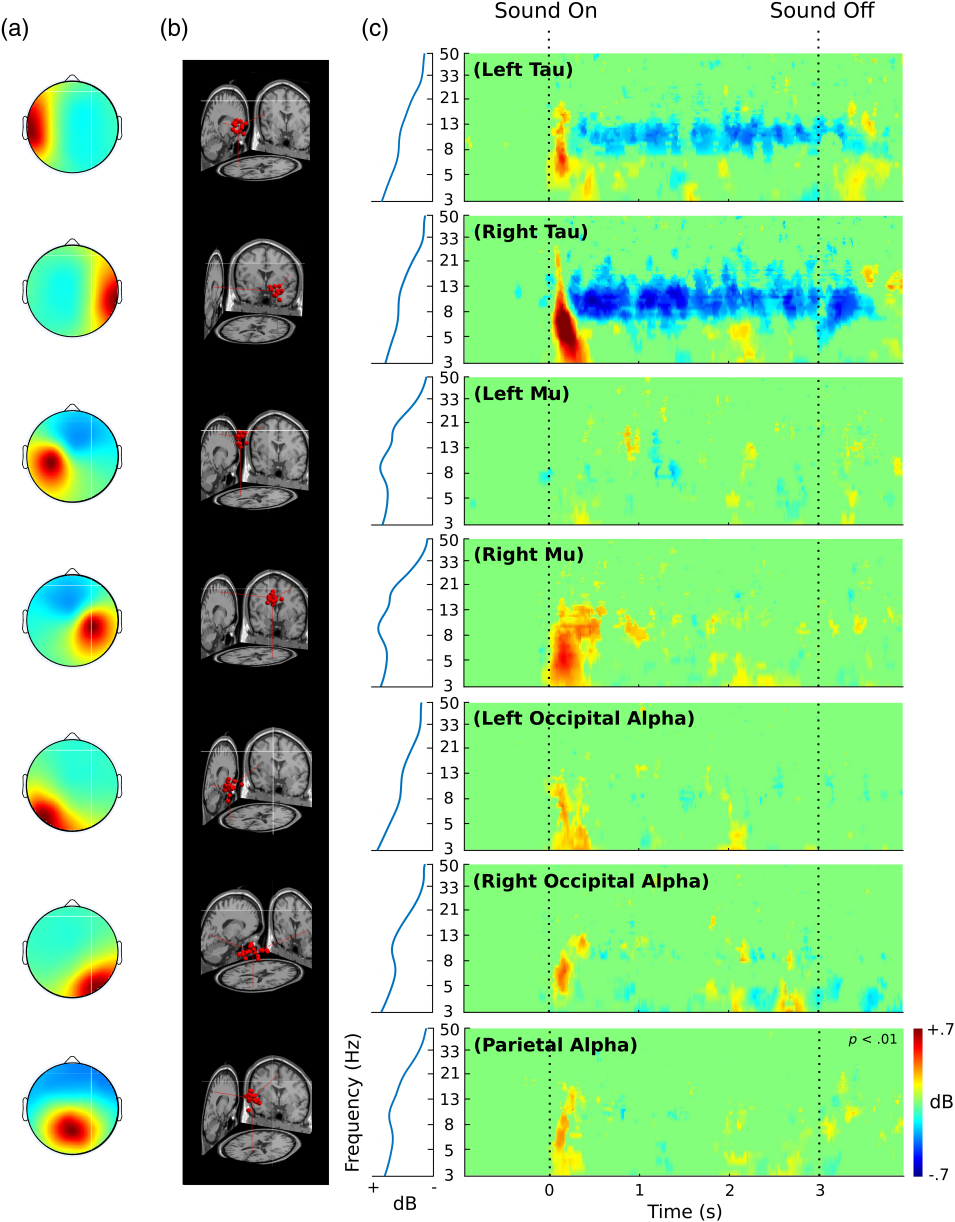
(a) Average scalp maps of independent component (IC) clusters corresponding to left tau, right tau, left mu, right mu, left occipital alpha, right occipital alpha, and parietal alpha. (b) Equivalent current dipoles for each cluster. Lines index the point of the cluster centroid on the X, Y, and Z axes in a template Montreal Neurological Institute (MNI) brain image with neurological convention (right side of image is right side of the brain). (c) Significance‐masked event‐related spectral perturbations (ERSPs) for different IC clusters. The leftmost portion of (c) shows the baseline spectrum for each cluster. Dashed vertical lines represent latencies of sound onset and offset.

The same procedure described above for selecting single IC candidates as a tau IC for each contributing subject was applied to the left and right mu rhythm IC clusters, the left and right occipital and central parietal alpha‐producing clusters. These clusters were also examined because they have been seen to produce strong alpha activity, and traditionally have scalp maps that overlap with, for example, the tau IC scalp maps shown in Figures 3 and 5. Average scalp maps for all these clusters and their modeled equivalent current dipoles are presented in Figure 6a,b, respectively. Significance masked ERSPs for each IC (*p* < .01) are shown in Figure 6c along with their mean baseline power spectra.

Both the left and right tau IC clusters show a significant alpha (~8–13 Hz) suppression relative to baseline during sound presentation. This relative alpha suppression sustains throughout the stimulation period. There are also transient spectral enhancements at a broad range of frequencies from the lowest computed frequency (3 Hz) extending up into the low beta range just after sound onset. While the other IC clusters also show similar transient power enhancements, none of the other clusters show sustained, significant alpha suppression, unlike the tau IC clusters. That is, tau rhythm suppression is not coincident with alpha suppression in other alpha‐producing IC clusters.

### 3.4 mAMICA model likelihood is related to tau IC alpha power

**FIGURE 7:**
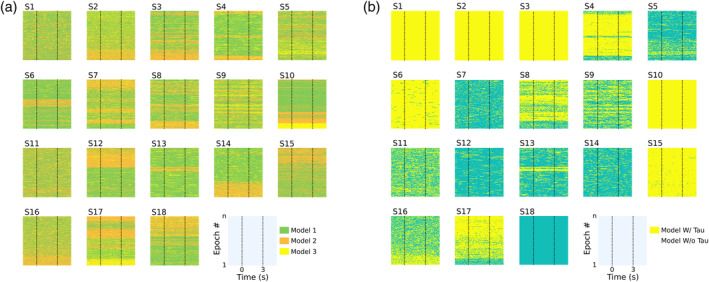
(a) Multi‐model adaptive mixture independent component analysis (mAMICA) most probable models for each subject over time. The y‐axes represent time within the dataset, first epoch (bottom) to last epoch (top). The x‐axes represent time point within epochs. Color represents the most probable model at each time. Individual subjects are labeled (S1–S18). (b) Same data as in (a), but separating models with at least one tau independent component (IC) from models with no identifiable tau IC.

Some important questions arise from the finding in Section 3.2 that mAMICA performed the best in regard to producing the greatest percentages of the sample with tau ICs. We first asked what aspects of the data led to nonstationarities. For instance, Hsu et al. (2018) found that during their continuous performance driving task the most probable mAMICA model, given the data, varied as a function of time‐on‐task (as did mean performance), with model switches typically occurring at transitions between “non‐drowsy” and “drowsy” performance states. In their data, this was revealed by plotting model probability as a function of task events and the time within the experimental session. Here, we take the same approach. Figure 7a plots the most likely model of the three mAMICA models over the entire session for each subject. The x‐axis indicates latency (in s) relative to onsets and offsets of the asynchronous tone trains. Individual trials are arrayed along the y‐axis. Color in the figure represents the most likely model (green = returned model 1, orange = model 2, yellow = model 3, model 1 generally accounting for more of the data than the other two models).

The aspect of this data that stands out most strongly is that in many subjects large swaths of the epochs appear to be associated with one or another model. For instance, S12 shows Model 1 being the most probable model for ~2/3 of the session. Near the last third of the experiment, Model 2 becomes more probable. Several other subjects show similar transitions (e.g., subjects 2, 3, 10, 14, 15, 16, and 18), while other subjects show transitions back and forth between models over the course of their session (e.g., subjects 4, 6, 7, 8, and 17). This replicates the findings from Hsu et al. (2018) who observed transitions between models occurring throughout a session in some of the paradigms they studied.

Figure 7b plots the same as Figure 7a, except we highlight whether an mAMICA model identifies a tau IC or not (at least 1 tau IC either left or right). Figure 7b can be cross‐referenced with Figure 7a to identify mAMICA models with a tau IC. This data shows the variability of tau IC presence across models in subjects. S1, S2, S3, and S10 show a tau IC for all 3 mAMICA models, while other subjects only show a tau IC for a portion of models, or no model at all (as is the case with S18). As with Figure 7a, this data does not reveal any consistent relationships with session time in regard to tau IC model probability. To determine whether tau model likelihood was related to alpha power in tau ICs, we asked whether the dynamics of alpha power for tau ICs reported in Figure 6 are related to the likelihood of a tau model as reported in Figure 7b.

This analysis was restricted to the 13 subjects who showed tau in only a subset of models. First, we recreated vectors of alpha power for each subject's lowest ranked tau component with a three‐cycle complex wavelet at 10 Hz. We then created a matched vector of the mean number of sample points within the 150 ms surrounding the center of each wavelet window in which a tau model from mAMICA was the most likely (cf. Figure 7b). This vector was baseline corrected by taking the percent relative number of sample points from the same baseline window as ERSPs (see Section 2.8).

**FIGURE 8:**
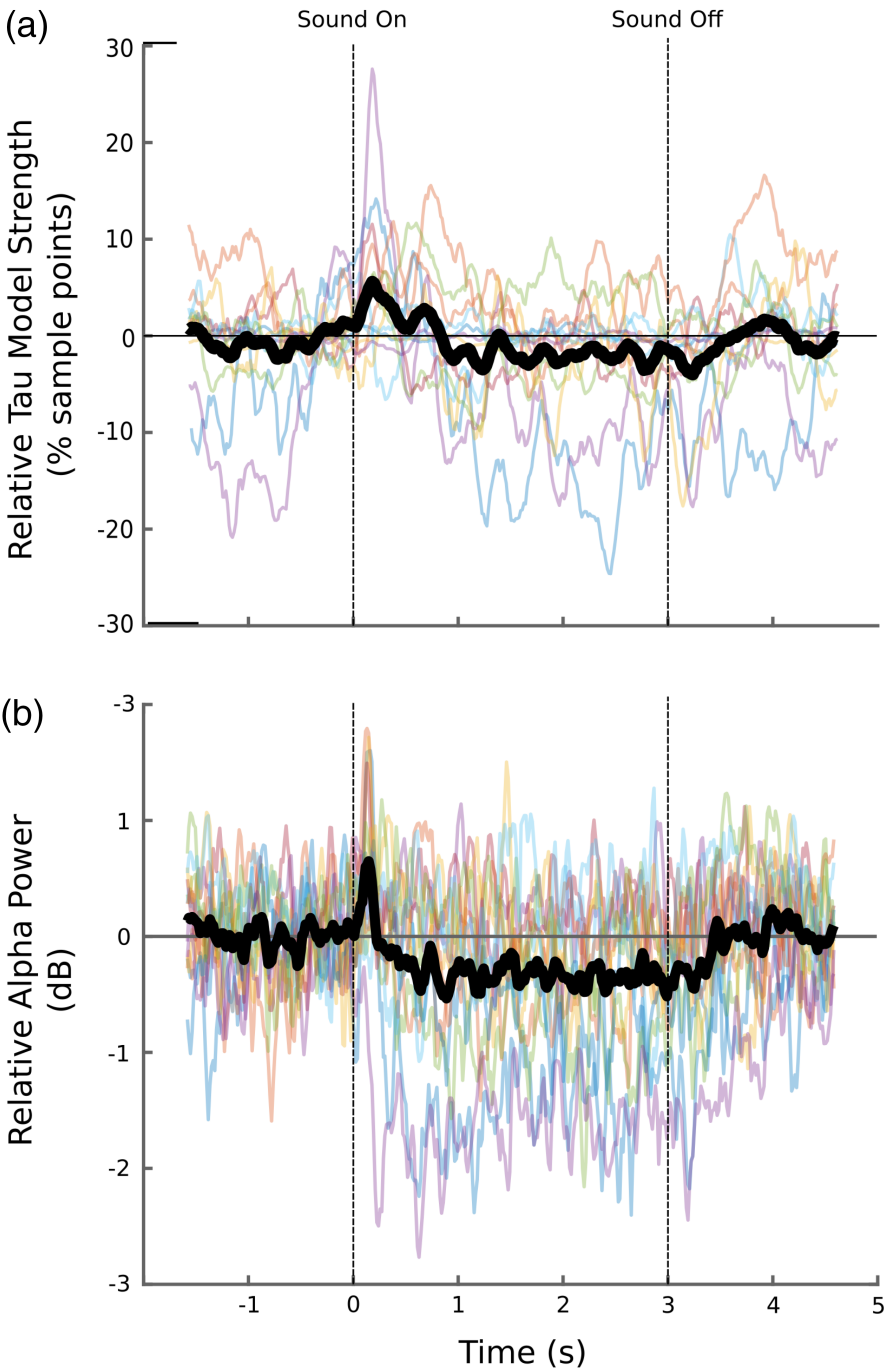
(a) Percent of sample points within each time‐frequency computation window in which a tau model from multi‐model adaptive mixture independent component analysis (mAMICA) was most likely. (b) Relative 10 Hz power for tau independent components (ICs). In both (a and b), thin colored lines represent individuals. Thick black lines represent the mean.

Figure 8 shows the individual and mean change in tau points relative to baseline (a), and individual and mean 10 Hz traces (b). From the plot panels it appears as though the likelihood of a tau model is related to the strength of alpha oscillations for tau ICs. The number of sample points containing a tau model goes up at those times where alpha oscillations are most powerful for tau ICs (e.g., immediately after sound onset). Likelihood is lowest at those points where alpha is low (e.g., during continuous sound presentation). We calculated Spearman's *rho* values for each subject to assess this relationship. A one‐sample *t*‐test assessing whether subjects' Spearman's *rho* values were significantly different from zero revealed significance, Mean *rho* = 0.20 (SD = .25), *t*(12) = 2.87, *p* = .014.

## 4 DISCUSSION

Though literature has established convincing evidence for auditory‐related tau rhythms using intracranial and MEG recordings, tau rhythms have been more elusive in EEG data. Several have suggested that ICA decomposition can be used as a means to examine tau apart from other stronger alpha rhythms present in the EEG. However, two main concerns have hindered the adoption of this approach. The first, is a sparsity problem wherein decompositions have returned a relatively low percentage of sample subjects contributing to IC clusters showing dynamics consistent with tau rhythms (~50% for Jenson et al., 2015 and Wisniewski et al., 2021). Our data here show that the right filter parameters and ICA model can increase the likelihood of identifying tau ICs in subject data. In our data, both left and right tau ICs were identified in >70% of the sample, with ~94% having at least one tau IC (i.e., left or right). This makes it more feasible to build hypotheses regarding tau rhythm ICs and to invest in studies that test these hypotheses. A second concern with an ICA‐based approach has been the possibility that tau ICs do not reflect tau as characterized in MEG and intracranial recordings, but rather reflect some other brain process (i.e., the distinctiveness problem). Here, we were able to demonstrate tau IC dynamics that show striking similarities to tau rhythm dynamics as seen in MEG and intracranial recordings. ICs of the tau type are localized along or near the superior temporal plane. They show alpha suppression during sound presentation while other ICs producing alpha rhythms do not.

### 4.1 Recommendations for analysis

Low‐pass filter settings did not have a significant impact on the likelihood of obtaining a tau IC from a subject, but high‐pass filter settings had a clear effect. Relatively low‐frequency passband edges (e.g., 0.1 Hz) were suboptimal. This has also been observed for other types of ICs (Klug & Gramann, 2021; Winkler et al., 2015). Relatively aggressive high‐pass filters using a passband edge frequency of up to 4 Hz performed better. One potential problem with using such aggressive high‐pass filters is that they can distort low‐frequency event‐related responses in the EEG and computed event‐related potentials (e.g., Duncan‐Johnson & Donchin, 1979; Tanner et al., 2015). Several have thus advocated using high‐pass filter settings well below those optimal for ICA decomposition (e.g., a 0.1‐Hz or lower half amplitude cutoff; Duncan et al., 2009; Luck, 2005). However, strict adherence to these recommendations will likely yield too few tau ICs for analysis. A reasonable compromise is to save a conservatively high‐pass filtered dataset before filtering the data with a more aggressive high‐pass filter. ICA can be run on the latter, then the trained ICA weights can be applied to the former. This procedure allows for the identification of usable IC processes while at the same time avoiding high‐pass distortions in data analysis. It may, however, carry some risk of IC mis‐assignment of low‐frequency activity in the data, should this be a focus of the research in question. Winkler et al. (2015) found that though high‐pass filtering up to ~1 Hz increased the number of near dipolar ICs returned by an ICA decomposition, increasing cutoffs too high (generally above 5 Hz) steadily decreased this number, likely because ICA identification of brain ICs requires low‐frequency information for spatial segregation of many commonly‐identified EEG sources. This is also why we did not test use of even stricter band‐pass filtering methods before running ICA.

The choice of ICA algorithm also impacted the likelihood of obtaining tau rhythm ICs. The ranking in our data were: mAMICA > AMICA > extended Infomax > fastICA. These results are in line with Delorme et al. (2012) who found that AMICA > infomax > fastICA in regard to producing stronger IC model “dipolarity” and mutual information reduction. Our data suggest that the use of fastICA is not likely to give researchers much advantage in the analysis of tau in EEG data (though with this algorithm the exact choice of parameters may affect the results). The main benefit of mAMICA is that it can adapt to non‐stationarities in the data. Regarding tau rhythms this could be especially important given previous MEG findings of tau disappearing during active listening (Keitel & Gross, 2016), and prominent tau appearance during drowsy states (Yokosawa et al., 2020). In our data, there was some suggestion that the strength of tau oscillations was associated with greater tau model likelihood (see Figure 8). However, we only had 13 subjects who showed a tau IC in only a subset of models. Determination of whether mAMICA works best because of its ability to pick up on transient tau bursts will require more subjects in a future study. It is also the case that the use of multiple models yields a larger number of brain‐related ICs than single model decompositions. This benefit has a trade‐off in that ICs from different models may not be maximally independent from each other, as each was trained on a data subset exhibiting some mAMICA‐detected differences with other model data subsets.

We did show that the ERSP dynamics of tau ICs and other IC types were different even when ICs taken for each subject were from different models (see Figure 6). Though caution is necessary when combining analysis of ICs across different mAMICA models, the benefits may outweigh this weakness. Certainly, there are other popular approaches to the isolation of brain sources that can be more problematic in this regard (e.g., group ICA—Janssen et al., 2020; PCA—Dien et al., 2003; classifier decoding—de Vries et al., 2021). This is because these approaches either ignore brain source temporal independence, or group subject data prior to model training (i.e., they ignore between‐subject variability in the patterns of tau source projection to the scalp).

Based on the current study, we expect that the use of high‐pass filters with passband edges between 2 and 4 Hz, followed by mAMICA decomposition, will yield more tau ICs for researchers than the adoption of rules of thumb (e.g., high‐pass filter cutoff of 0.1 Hz; Luck, 2005) or automatic use of common methods (e.g., fastICA). Much like how filter parameters for measuring ERP components have been optimized over time, continuing work in this domain will help to optimize these choices. If one wishes to use caution in adopting the more complex, less common mAMICA approach to ICA decomposition, single‐model AMICA may remain a more useful alternative than fastICA or even extended infomax (cf. Delorme et al., 2012). However, it is promising that when some subjects only show tau in a subset of models, mAMICA appeared to at least partially identify the tau model based on the appearance of relatively high oscillatory power in the alpha range (see Figure 8). Thus, mAMICA could be a promising means to identify and measure tau rhythms in EEG when non‐stationarities in subject brain dynamic state lead to tau only being detectable for brief periods of time (e.g., near sound onsets and offsets, during more alert or drowsier data periods, etc.).

### 4.2 Why use an ICA decomposition approach to analysis of tau rhythms?

MEG and intracranial recording methods are more restrictive than EEG recording in regard to access, cost, and range of employable paradigms. While MEG and particularly intracranial data will continue to provide value in regard to spatial certainty of tau dynamics, ICA decompositions of EEG data can be used in complementary studies with more variety in paradigms and subject populations. To support this supposition we briefly highlight two current areas of study that could benefit from an ICA approach to tau rhythm analysis in EEG: tinnitus research and research on alpha dynamics and attention.

Tinnitus is a disorder associated with the conscious sensation of sound without a corresponding external input (Baguley et al., 2013). Tinnitus has high prevalence with estimates of about 10% of adults experiencing it (Davis & El Rafaie, 2000). While most patients are not troubled by it, others find it especially disturbing (Baguley et al., 2013). MEG studies have revealed that tinnitus sufferers have an abnormally low amplitude resting tau rhythm that is related to the severeness of symptoms (Güntensperger et al., 2020; Hartmann et al., 2014; Müller et al., 2013; Weisz et al., 2011). Neurofeedback to increase cortical alpha activity can decrease tinnitus symptoms (Güntensperger et al., 2020; Hartmann et al., 2014). For instance, Hartmann et al. (2014) gave patients neurofeedback aimed to increase summed EEG alpha power in the scalp channel data. This led to increased power of the tau rhythm, as measured by post‐neurofeedback MEG, and a significant reduction in tinnitus symptoms.

However, neurofeedback‐related decreases in symptoms often still leave tinnitus at a level that is distressing (Güntensperger et al., 2020). It is possible that instead of relying on a measure of the whole data, or at a single electrode channel, for control of neurofeedback, one might tau IC power measures computed online. This would allow a more specific focus on the tau rhythm, and could potentially yield stronger changes to the tau rhythm and tinnitus symptoms. This would help tinnitus researchers achieve a level of symptom reduction that is clinically relevant, while also being relatively low‐cost to implement. For example, in a single‐subject trial, Onton and Makeig (2006) showed that basing neurofeedback on the level of mu rhythm in a left mu‐producing IC not only allowed some measure of neurofeedback control over its level but remarkably, in the fourth such session produced that effect without concurrent effect on the power of the posterior alpha rhythms (see also Delorme & Makeig, 2003; Makeig et al., 2000). Using an ICA‐based measure of tau, it would also be easier to characterize other potential neurofeedback treatments' effects on the tau rhythm by measures of tau specifically. The wide‐spread availability and low‐cost advantages of EEG recording would allow more subjects to be studied, and more researchers (and, if successful, clinicians) to perform the work.

Attention has been closely linked to the level of alpha activity in the EEG. Though most of this work has examined visual or somatomotor alpha rhythms (for review, see Klimesch, 2012; Klimesch et al., 2007), this has also been shown in auditory studies. For instance, numerous works in recent years have focused on using alpha power as an index of “listening effort” (for review, see Francis & Love, 2020). Several have found that greater effort is associated with increased total alpha power at electrode locations over posterior scalp (e.g., Obleser et al., 2012; Wisniewski et al., 2017; Wöstmann et al., 2017). The presumed link to attention is that alpha increases in nonauditory regions as a means to inhibit processing, and thereby distraction produced by sensory experiences processed in those areas, in turn focusing processing to more relevant brain networks (cf. Jensen & Mazaheri, 2010; Van Diepen et al., 2019).

Though examination of these alpha enhancements in nonauditory regions has been useful in characterizing how various stimulus and cognitive manipulations impact attentional demands during listening (for review, see Francis & Love, 2020), this is a rather indirect way of examining auditory processing. A more direct route would involve characterizing how specific suppression of tau rhythm in tau ICs is related to auditory attention. A hypothesis consistent with the alpha rhythm literature is that, as alpha activity is an inhibitory brain mechanism (Bastiaansen et al., 2001; Jensen & Mazaheri, 2010; Klimesch, 2012; Klimesch et al., 2007; Van Diepen et al., 2019; Weisz & Obleser, 2014), when one needs to focus on the auditory environment, alpha suppression should be seen in tau rhythm ICs. Some studies have suggested that tau suppression may exist in the presence of occipital or somatomotor alpha activity enhancement (e.g., Dimitrijevic et al., 2017; Wisniewski et al., 2021), but this has not often been observed in the same study (although, see Mazaheri et al., 2014; Wisniewski & Zakrzewski, 2023).Examination of tau ICs in the context of concurrent dynamics in other ICs could help in extending this hypothesis to include the auditory modality. For example, during increased auditory (vs. visual) attention alpha enhancement should be seen in somatomotor ICs while alpha suppression is observed in tau ICs.

### 4.3 Future work in the development of ICA approaches to tau rhythm analysis

Dipole modeling of the obtained tau ICs showed cluster centers in or near left and right supramarginal gyri. This does not exactly overlap with recent intracranial recordings of the tau rhythm (Billig et al., 2019), although there is MEG work suggesting that tau rhythms have additional sources in the supramarginal gyri (van Dijk et al., 2010). The intracranial work found the strongest resting tau rhythms in secondary auditory cortical areas. Here, we recorded individual electrode locations for each subject, then warped these locations to a standard head model before fitting. This approach is generally effective, but can also be associated with errors in equivalent dipole localization (Akalin Acar & Makeig, 2013). Adding to the problem, simulation data suggest that localization errors are larger in the temporal lobe compared to other lobes of the cerebral cortex, with errors tending toward locations superior to the simulated temporal sources (Akalin Acar & Makeig, 2013). Future research should use individually fit forward head models rather than generic head models. This could be done by obtaining MR head image scans for each subject, then building a forward model from those images (Akalin Acar & Makeig, 2013). Dipole modeling using such individualized head models has been shown to improve the accuracy of IC source localization (Akalin Acar & Makeig, 2013). Using this approach, we might establish stronger confidence that the tau ICs we observe reflect the tau rhythms observed with intracranial and MEG methods.

Future work should also evaluate the relationships between tau IC dynamics and behavior. While use of the passive presentation task here maps closely to the research that established the suppression of tau rhythms during auditory presentations, it does not allow examination of brain–behavior relationships. There are several predictions coming from theories of alpha oscillations as an inhibitory mechanism in the brain (for review, see Weisz & Obleser, 2014) that could be evaluated with tau ICs. One prediction is that the accuracy of auditory judgments should relate to the prestimulus tau IC alpha power such that less prestimulus alpha power would predict better listening performance. It might also be that the phase of prestimulus tau rhythms at sound onsets should affect listening performance, with correct and incorrect trials exhibiting opposing phase angles at sound onsets (cf. Hansen et al., 2019). Still another possible prediction is that tau enhancement, rather than suppression, should relate to performance at moments when an ongoing auditory stimulus stream needs to be ignored (Hartmann et al., 2014). Studies along these lines will go a long way in establishing whether and how tau rhythms relate to behavior, and whether the relationships parallel those associated with other brain alpha‐band rhythms.

## 5 CONCLUSIONS

We have demonstrated that an ICA decomposition approach to analysis of EEG data can be a useful means by which to isolate and analyze tau rhythms. This opens possibilities for studying auditory‐related alpha rhythms (tau) as visual and somatomotor (mu) alpha rhythms have been studied in the past using EEG recording. We here have demonstrated that MEG or intracranial recording are not necessary for examination of cortical tau activity. Further, we have shown that ICA decomposition performed using optimal algorithm selection and preprocessing filters can dramatically increase the probability of detecting tau rhythms in suitable EEG data.

## CONFLICT OF INTEREST STATEMENT

None of the authors have conflicts of interest to disclose.

## Data Availability

The data that support the findings of this study are available from the corresponding author upon reasonable request.

## Footnotes

^1^
Readers should also note that left and right temporal ICs have also been used to examine auditory steady‐state responses (ASSRs; e.g., Farahani et al., 2017; Makeig et al., 1997).
